# A probabilistic framework for windows of opportunity: the role of temporal variability in critical transitions

**DOI:** 10.1098/rsif.2022.0041

**Published:** 2022-05-04

**Authors:** Jim van Belzen, Gregory S. Fivash, Zhan Hu, Tjeerd J. Bouma, Peter M. J. Herman

**Affiliations:** ^1^ Department of Estuarine and Delta Systems, Royal Netherlands Institute for Sea Research (NIOZ), 4401 NT Yerseke, The Netherlands; ^2^ School of Marine Sciences, Sun Yat-Sen University, and Southern Marine Science and Engineering Guangdong Laboratory (Zhuhai), Zhuhai, People's Republic of China; ^3^ Guangdong Provincial Key Laboratory of Marine Resources and Coastal Engineering, Guangzhou, People's Republic of China; ^4^ Pearl River Estuary Marine Ecosystem Research Station, Ministry of Education, Zhuhai, People's Republic of China; ^5^ Faculty of Geosciences, Department of Physical Geography, Utrecht University, 3508 TC Utrecht, The Netherlands; ^6^ Department of Hydraulic Engineering, Delft University of Technology, 2628 CN, Delft, The Netherlands; ^7^ Unit of Marine and Coastal Systems, Deltares, 2600 MH, Delft, The Netherlands

**Keywords:** critical transitions, establishment, noise-induced transition, population and community dynamics, stable state, temporal variability

## Abstract

The establishment of young organisms in harsh environments often requires a window of opportunity (WoO). That is, a short time window in which environmental conditions drop long enough below the hostile average level, giving the organism time to develop tolerance and transition into stable existence. It has been suggested that this kind of establishment dynamics is a noise-induced transition between two alternate states. Understanding how temporal variability (i.e. noise) in environmental conditions affects establishment of organisms is therefore key, yet not well understood or included explicitly in the WoO framework. In this paper, we develop a coherent theoretical framework for understanding when the WoO open or close based on simple dichotomous environmental variation. We reveal that understanding of the intrinsic timescales of both the developing organism and the environment is fundamental to predict if organisms can or cannot establish. These insights have allowed us to develop statistical laws for predicting establishment probabilities based on the period and variance of the fluctuations in naturally variable environments. Based on this framework, we now get a clear understanding of how changes in the timing and magnitude of climate variability or management can mediate establishment chances.

## 1. Introduction

In many harsh environments, the establishment of young organisms is the most difficult phase of the life cycle [1–3]. In arid environments, for example, the successful establishment of trees is episodic and requires a year of exceptional rainfall [4,5] or a period of low consumption pressure due to unusually low grazer abundance [4,6]. In coastal wetlands, such as tidal marshes and mangrove forests, the episodic establishment of vegetation has been linked to rare periods of mild hydrodynamic exposure [7,8]. Moreover, the episodic recruitment of shellfish reefs has been suggested to be the result of unusually low predation pressure by crustaceans [9–11]. Such time intervals of benign conditions in which organisms suddenly establish have been coined windows of opportunity (WoOs) [4,12].

It has been suggested that the observed episodic establishment dynamics, typical for many harsh environments, can be explained as noise-induced critical transitions between two alternative stable states [7]. Under this framework, environmental variability is vital in creating periods of unusually benign conditions. Such windows allow young organisms that are unsuited to the average environmental condition to reach a stable existence by rapidly growing and developing sufficient tolerance to that environment [7,13]. Such establishment events are thus the result of the fact that, while long-term environmental conditions may on average remain stationary (i.e. no increasing or decreasing trends in the long-term average or variability of the stressor), environments constantly vary on shorter timescales. Without such environmental variability no transitions can occur [7,8,14]. To date, the role of the period (i.e. determining the intermittence of benign and harsh conditions) and variance (i.e. the magnitude of the fluctuation) of the temporal variability in such noise-induced transitions that ultimately lead to the emergence of ecosystems remains a topic that is ill explored. Hence, this hampers the understanding and prediction of organism dynamics in variable environments [5,15].

In this paper, we further develop the concept of WoO into a coherent theoretical framework to systematically explore such episodic dynamics and derive establishment probabilities. We reveal that for young organisms to exploit windows of benign conditions, sufficiently large and temporally correlated environmental variability is the most critical requirement. Yet, for the organisms to be able to turn such benign windows into opportunities for establishment they need to exhibit stage-dependent tolerance (as the result of, e.g. physiological maturation or density-dependent feedbacks) and the ability to grow and develop their tolerance swiftly. We support our argumentation by (1) developing a theoretical framework based on organisms developing in a regular dichotomous environment. (2) Explaining how correlated variability (i.e. noise that has a non-negligible correlation time) shapes the statistical probability to allow critical transitions towards establishment of these organisms, and eventually the ecosystem. We then show (3) that the conceptual framework also holds for continuous environmental signals. Finally, (4) we discuss the consequences of these insights for the interpretation of stability and recoverability to real-world ecosystems and how establishment success can be mediated to facilitate management and restoration.

## 2. Theoretical framework for windows of opportunity

To establish, organisms need to rapidly gain tolerance against hostile conditions of the environment it is embedded within. Due to growth and development, organisms physiologically mature or can invoke density-dependent feedbacks that improve their environmental tolerance. Consider for instance a plant that develops a root system over time that allows it to reach deeper groundwater layers, increasing its tolerance to drought episodes [5,16] or prevent them from uprooting in intertidal sediments due to tidal current and wave action [8,12,17]. Moreover, becoming larger is often a good way to escape from predation or herbivory: trees that grow tall enough may escape the herbivory of their foliage [18]. Bivalves are also known to become less vulnerable to predation by crustaceans once a size threshold is surpassed [10]. Becoming larger can even increase an organism's ability to tolerate toxic conditions [19,20]. In the above examples, the size or biomass of an organism is thus directly linked to its tolerance, especially in the earliest stages of life, underlining the key concept of stage-dependent tolerance.

**Figure 1.**
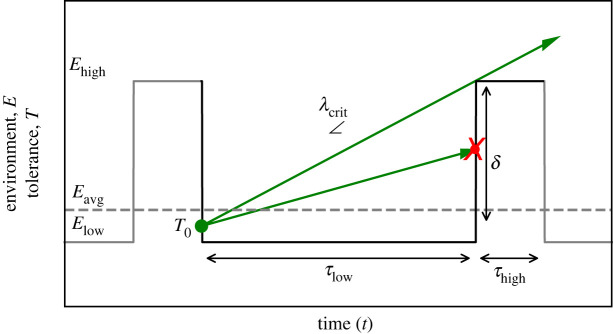
Conceptual framework for WoO. Organisms develop tolerance (green arrows) in a regular dichotomous fluctuating environment (black and grey). The organism needs to overcome tolerance threshold *δ* to establish. The inclination of the upper green arrow depicts the critical growth rate *λ*_crit_ just high enough to be successful. For further explanation, see text.

To develop an intuitive understanding of how individual young organisms can establish in such a hostile environment and to encapsulate the concept of stage-dependent tolerance in the theoretical framework, we explore a model (figure 1) in which a young organism is trying to establish within an environment *E* that fluctuates as a regular dichotomous signal between a low and high value, *E*_low_ and *E*_high_, respectively (statistical properties of the regular dichotomous signal are given in electronic supplementary material, note S1). The environment is stationary at a longer timescale, characterized by periodic transitions between *E*_low_ lasting for a fixed time window *τ*_low_, and *E*_high_ lasting for *τ*_high_. The state of the young organism is defined by its tolerance *T* to this environmental stressor. From inception (that is e.g. birth, germination, attachment or transplantation), the organism starts developing from an initial tolerance *T*_0_ with a growth rate *λ* (green lines, figure 1). If the organism's tolerance *T* to the environment *E* has not developed high enough before the environment returns to the harsh condition *E*_high_, it dies instantaneously (as *T < E* at *τ*_low_, lowest green arrow with red cross in figure 1 is a failing individual as it developed tolerance at a too slow rate). If the tolerance *T* is developed sufficiently (*T > E*_high_), the individual establishes. Hence, the individual needs to overcome a threshold *δ* = *E*_high_ *− T*_0_ to establish by developing tolerance with a growth rate *λ ≥ λ*_crit_. Considering this framework, we can identify four types of regimes from the perspective of the young organism: (i) a benign environment in which *T*_0_ *≥ E*_high_ > *E*_avg_; (ii) a benign environment in which *E*_high_ > *T*_0_ *≥ E*_avg_; (iii) a harsh environment in which *E*_avg_ > *T*_0_ > *E*_low_; and (iv) a harsh environment in which *E*_avg_ > *E*_low_ > *T*_0_*.* In the benign environment (i) establishment can always occur, while in the harsh environment (iv) it never does. In situation (ii), environmental variability will destroy possibilities for establishment rather than creating them. Thus, our interest and focus of this paper is mainly on the third set (iii) of conditions (figure 1) in which establishment is only possible if tolerance is increased enough after inception (e.g. due to growth) before the environment switches to harsh conditions *E*_high_.

From this simple conceptual framework based on the regular dichotomous signal (figure 1), we can easily derive (see electronic supplementary material, note S2) that the critical growth rate *λ*_crit_ (i.e. minimum rate) at which an organism needs to develop tolerance to be able to establish successfully is

2.1 $$\lambda_{\mathrm{crit}}=\frac{\delta }{\tau_{\mathrm{low}}}.$$

This relation immediately reveals how the period *τ* and variance *σ*^2^(*E*) of the environmental fluctuations are key parameters in determining the growth rate requirements for establishment. The threshold *δ* is related to the variance *σ*^2^(*E*) which the organism is exposed to. More specifically, *δ* is a system-specific parameter determined by the difference between the initial tolerance *T*_0_ and the maximum environmental condition *E*_high_. The type of development of tolerance over time (e.g. linear or exponential growth) is a key factor that determines the organism's sensitivity to fluctuations in environmental conditions (electronic supplementary material, figure S1, note S4). Organisms developing tolerance exponentially can be less sensitive to changes in the variance *σ*^2^(*E*) compared to organisms developing tolerance linearly (electronic supplementary material, figure S1, note S4). The period *τ*_low_ of the fluctuations reflects the intrinsic timescale (i.e. temporal correlation) that needs to be matched by the organism independent of growth type. Hence, the critical growth rate *λ*_crit_ is directly proportional to the inverse of the benign time window *τ*_low_

2.2 $$\lambda_{\mathrm{crit}}\propto \frac{1}{\tau_{\mathrm{low}}}.$$

Fulfilling criterium *λ* ≥ *λ*_crit_ implies that a time window of benign conditions *τ*_low_ becomes a WoO, in which establishment will be successful. Environmental variability thus needs to have a long enough time windows *τ*_low_ for establishment to be possible.

**Figure 2.**
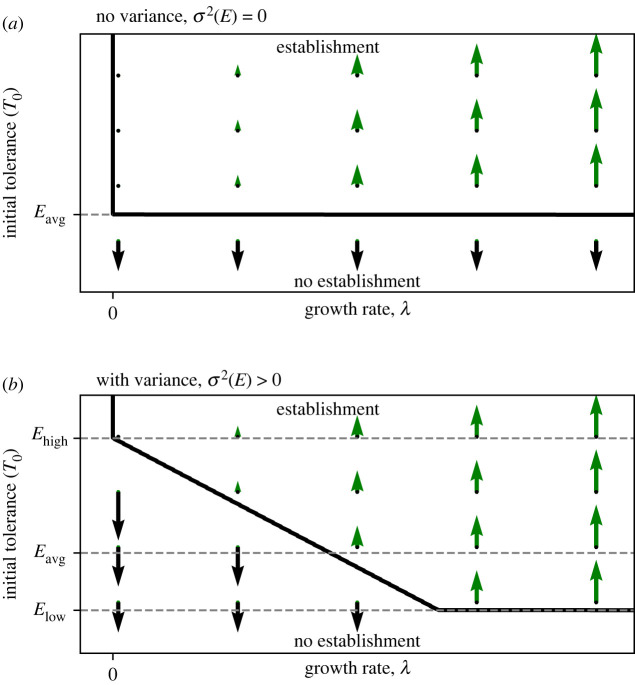
General bifurcation diagrams. (*a*) The case of no environmental variance, and (*b*) with environmental variance, i.e. with a regular dichotomous fluctuating environment (figure 1). The bold arrows indicate the growth rate and direction towards which the organism develop tolerance, which is either towards establishment (upward green arrows) or no establishment (downward bold black arrows). The size of the upward green arrows indicates the growth rate *λ* of change in tolerance allowing the organism to establish and stably exists. The thick black line represents the unstable equilibrium between either state, i.e. the null-isocline.

From this framework, we can furthermore understand that the existence of sufficient environmental variability is key to create opportunities to get into stable existence. Without sufficient variance (i.e. *σ*^2^(*E*) = 0, thus *E*_avg_ = *E*_low_ = *E*_high_, figure 2*a*) WoOs (or non-opportunity) do not exist (i.e. the system collapses to regimes i and iv). Instead, the only pathway to establishment is for an organism to have an initial environmental tolerance *T*_0_ > *E*_avg_. Sufficiently large variance (i.e. *σ*^2^(*E*) < 0 in which *E*_low_ < *T*_0_) must be introduced to the system so that benign intervals will appear in which an organism of tolerance *E*_high_ > *T*_0_ ≥ *E*_low_ would have the potential to establish (figure 2*b*). Once the variance *σ*^2^(*E*) is large enough, noise-induced transitions towards establishment can occur for organisms with a growth rate exceeding *λ*_crit_. The null-isocline (thick black line in figure 2*b*) reflects what value of *λ*_crit_ corresponds to what value of *T*_0_ (figure 2*b*). Here we can see there is a range of growth rates *λ* that allow establishment if *T*_0_ < *E*_avg_ (regime iii). Yet, at the same time environmental variability also poses a threat to organisms by increasing the distance between *E*_low_ and *E*_high_, resulting in the need for a critical growth rate *λ*_crit_ even if *T*_0_ ≥ *E*_avg_ (regime ii). Therefore, an organism that can establish in the benign environment without temporal variability (figure 2*a*) must have a sufficiently high growth rate *λ* in order to overcome the impending switch to a hostile condition *E*_high_ in the case of the environment with variability (figure 2*b*).

Summarizing, from the established conceptual framework, we developed a sound intuitive understanding of when WoO can occur. It is revealed that the understanding of the intrinsic timescales of both the organism developing tolerance to the environment and the environmental variability itself is key to understand if young organisms can or cannot establish in a hostile environment. It further highlights the key role that environmental variability plays in the occurrence of transitions between unestablished and established states. Specifically, it reveals that (1) without environmental variability, either no establishment can occur, or establishment is always possible; (2) benign time intervals must be long enough for organisms, whose growth rates are intrinsically limited, to be able to take advantage of them. Based on these insights, we can now further develop a probabilistic framework.

## 3. Statistical regularities in random environments

**Figure 3.**
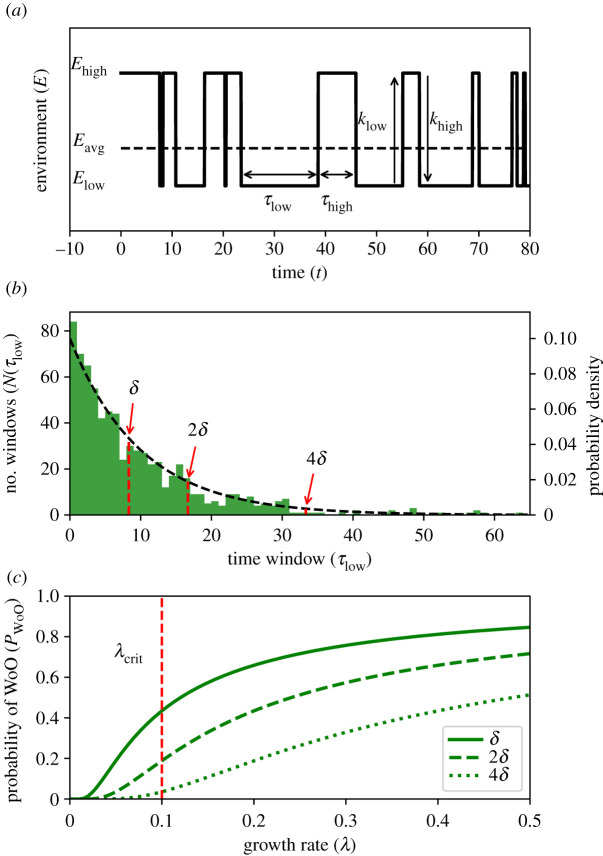
Statistical regularities derived from dichotomous Markov noise. (*a*) Example of a dichotomous Markov noise signal. The environmental signal fluctuates stochastically between a low and a high condition (*E*_low_, *E*_high_). The rates of transition (*k*_low_, *k*_high_) determine how long the environment remains in one of either conditions (waiting times *τ*_low_ and *τ*_high_). (*b*) The occurrence of benign time windows *τ*_low_ in one simulated time series (green bars) and the expected probability density based on long-term stationary dynamics of the signal (dashed line). At *λ*_crit_ = 0.1, the minimum window length required to have opportunity to establish depends on the threshold *δ*. At higher thresholds larger time windows are required (red arrows). The corresponding probability for WoOs to occur can be derived as the cumulative probability beyond this point. (*c*) Probability of WoO occurence *P*_WoO_ as a function of the growth rate *λ* for different values of *δ*. At too low growth rates, no establishment can occur. Here, the probabilities at *λ*_crit_ (dashed red line) correspond with the arrows in (*b*). For the example, a DMN signal of 10 000 time steps, with: *E*_low_ = 0, *E*_high_ = 1, *k*_low_ = 0.1, *k*_high_ = 0.2 and dt = 0.01 was used. *T*_0_ = 1/6 which makes *δ* = 5/6.

Now we have set up the theoretical framework for WoO, we can apply this to a more variable environmental signal in order to derive statistical rules and explore the role of environmental variability more systematically. To focus on understanding how environmental variability determines the probability of WoO to occur in variable environments, we first consider the simple dichotomous Markov noise (DMN) signal (figure 3*a*, statistical properties of DMN signal are given in electronic supplementary material, note S1). Like the regular dichotomous signal, the environment can only be in a high or low condition (*E*_low_ and *E*_high_). However, the length of the time windows (*τ*, or ‘waiting time') is no longer constant, but varies stochastically. The transition from one to the other condition depends on the transitions rates, *k*_low_ and *k*_high_. The mean benign window size is inversely related to the transition rate, thus 〈*τ*_low_〉 = 1/*k*_low_. As a result, DMN can capture the finite correlation time (i.e. the time at which the temporal autocorrelation *ξ* > 1*/e*) of a noise signal (i.e. coloured noise) very well and in a simple fashion. The finite correlation time is proportional to the approximate period 〈*τ*〉 of the environmental signal (i.e. the average period until/before the signal returns to the original position after an excursion). Furthermore, as we will keep 〈*τ*_high_〉 constant throughout the analysis and focus on varying 〈*τ*_low_〉 only, we therefore can use the approximate period 〈*τ*〉 to refer to the temporal aspect of the environmental variability throughout the remainder of this work. It furthermore makes it easier to study the interplay of the intrinsic timescale of the DMN and the timescale of the establishing organisms. It has been shown before that such interactions can lead to non-trivial effects such as multistability and hysteresis [21]. Thus, using the DMN signal, we can first focus on analytically solvable situations before we go into more complicated continuous environmental signals.

The probability of a time window with a specific length occurring in the stationary DMN signal corresponds to an exponential distribution as (figure 3*b*)

3.1 $$P(\tau_{\mathrm{low}})=k_{\mathrm{low}}\exp(-k_{\mathrm{low}}\tau_{\mathrm{low}}).$$

From proportionality relation (2.2), we can derive the critical time *τ*_crit_, i.e. the shortest time window required to turn a window into an opportunity. This critical window size *τ*_crit_ is basically determined by the critical growth rate *λ*_crit_ and the threshold *δ* that needs to be surpassed. Thus, the cumulative probability of all windows equal to or larger than this critical windows size determines the probability of a WoO *P*_WoO_ (figure 3*c*). This probability can be obtained as

3.2 $$P_{\mathrm{WoO}}=\exp(-k_{\mathrm{low}}\tau_{\mathrm{crit}}).$$

Based on equation (3.2), we systematically explore the consequences on the probability of WoO occurring. First, we focus on the environmental threshold *δ*, which can be increased due to a higher harsh environmental condition *E*_high_ (i.e. larger variance *σ*^2^(*E*) ∝ *E*_high_ − *E*_low_) or lower initial tolerance *T*_0_. When keeping the growth rate constant (e.g. *λ* = 0.1, red arrows in figure 3*b* and red dashed line figure 3*c*), a larger threshold *δ* requires increasingly larger critical window sizes *τ*_low,_ and these are less likely to occur (red arrows in figure 3*b*). Increasing the variance *σ*^2^(*E*), or lowering *T*_0_, thus consequently requires the organism to grow at a higher rate to acquire the same probability for establishment (figure 3*c*).

When focusing on the gradual increase of the growth rate (e.g. as would occur along a gradient from high to low physiological stress), the model reveals that at growth rates that are too low, the WoO (*P*_WoO_) becomes highly unlikely, but not nil (figure 3*c*). Yet, above a critical growth rate, the WoO for establishment suddenly becomes much more likely and rises with further increasing growth rate towards the asymptote at *P*_WoO_ = 1. The critical growth rate at which the probability for a WoO significantly improves depending strongly on the height of the threshold. Hence, the critical growth rate required for significant establishment chances increases proportionally with increasing environmental variance *σ*^2^(*E*).

The role of changing temporal autocorrelation (i.e. the period) can be revealed by changing the transition rate *k*_low_ (not shown). Increasing the transition rate *k*_low_ cuts the average window length (i.e. whitening of environmental signal *E*) and vice versa decreasing the transition rate *k*_low_ increases the average window length (i.e. reddening of *E*) and therewith the probability of establishment. Thus, reddening of the fluctuating signal increases the probability that a WoO will occur. Whitening of the environmental signal (i.e. approaching complete random noise, thus a fluctuating environment with neglectable temporal autocorrelation length) on the other hand will result in a nonlinear decrease of probability for a WoO to occur (i.e. sharp drop in *P*_WoO_). Generally, this agrees with recent work showing climate reddening increases the chances of critical transitions to occur [22].

**Figure 4.**
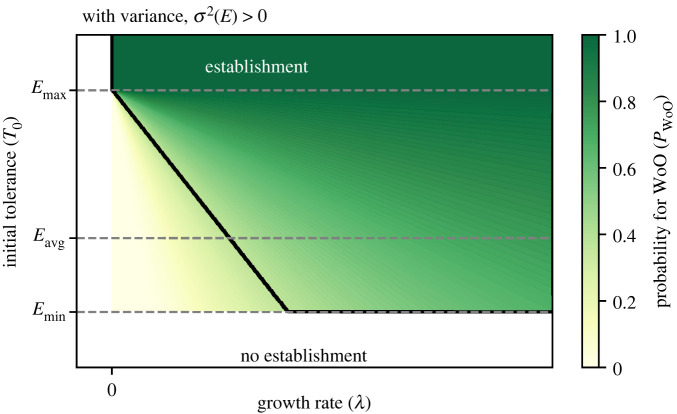
Generic establishment probability diagram in case of dichotomous Markov noise. For reference, the thick black line represents the unstable equilibrium (i.e. where the establishment chance shifts from 0 to 1) in case of a regular dichotomous signal (figure 1*c*).

Overall, the probabilistic framework can be summarized in a generic probability diagram (figure 4). This diagram is in essence similar to the bifurcation diagram (figure 2*b*, and therefore plotted for reference as the black line in figure 4). Yet, the strict dichotomous outcomes are now replaced with the probabilities of a WoO occurring. For example, at any given initial tolerance *T*_0_ with corresponding critical growth rate *λ*_crit_ specific for an environment with approximate period 〈*τ*〉, where it just crossed the threshold (thus *P*_WoO_ = 1) in the regular dichotomous case (black line from figure 2*b* in figure 4), the probability *P*_WoO_ of WoOs to occur is always 1/*e* (approx. 0.368). Thus, here WoOs for establishment occur in the DMN on average approximately once every third ‘cycle' the environmental signal is going through. This probability diagram for *P_*WoO*_* is not specific for a unique parameter set and can be drawn for any DMN signal and can be used to estimate the probability of establishment *P*_est_ (see electronic supplementary material, note S5). Yet, it is unclear how generic this diagram is beyond the realm of DMN signals, as insights derived from DMN signals might be too simplistic and overestimate establishment chances for more complicated continuous signals such as found in nature. Fortunately, we can now see if these statistical patterns are holding for other (non-dichotomous) environmental signals based on the developed statistical framework, which is the focus of next section.

## 4. Establishment in continuous environments

Thus far, we have derived understanding about how variance characteristics (i.e. period and variance) determine the occurrence of WoOs based on very simple dichotomous signals, as such signals are easy to understand and analytically tractable. However, most environmental signals are much more complicated. We therefore demonstrate next that the statistical rules derived remain largely intact when expanding the analysis to continuous environmental signals as can be encountered in real natural systems. To do so, we study how the occurrence probabilities of WoOs change as the result of changes in period and variance of temporally correlated Gaussian noise.

**Figure 5.**
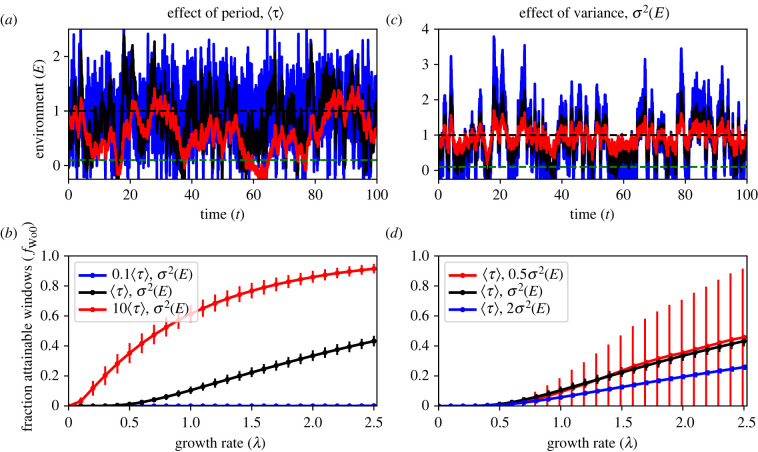
Statistical regularities derived from Ornstein–Uhlenbeck noise. (*a*,*b*) Variance *σ*^2^(*E*) is fixed and the approximate period 〈*τ*〉 differs a factor 10. (*c,d*) Approximate period 〈*τ*〉 is fixed and the variance *σ*^2^(*E*) differs a factor 2. (*a,c*) One hundred-time step subsections of the OUN signals created from the same sequence of random numbers. The environmental signal fluctuates stochastically around average *μ* (dashed black line). (*b,d*) Numerical results revealing the fraction attainable windows *f*_WoO_ as a function of the growth rate *λ*. For the numerical simulations, we used 1000 time steps which we replicated with 1000 synthetic datasets (see electronic supplementary material, note S6 for further model details) with default settings: *μ* = 1, 〈*τ*〉 = 1, *σ*^2^(*E*) = 0.5 and d*t* = 0.01 was used. Error bars indicate the interdecile range (10–90% of the simulations) and are slightly shifted for clarity.

Correlated Gaussian noise signals, as the results of the Ornstein–Uhlenbeck process (see electronic supplementary material, note S6 for details and figure 5*a,c* for example signals) have been shown to reflect many characteristics of complex natural signals (e.g. [21,23]). In this section, we numerically solve how organisms can establish in environmental signals constructed based on Hasselmann [24] variation of the Ornstein–Uhlenbeck noise (OUN). Changes in the temporally correlated signal *E* have an approximate period 〈*τ*〉 becoming red noise in case *τ* is sufficiently large

4.1 $$dE=\left(1\text{–}\frac{1}{\left\langle \tau \right\rangle }\right)(\mu -E)\;dt+\varepsilon R,$$

in which *R* is a random number drawn from a standard normal distribution with average nil resulting in the excursion of the signal *E* from its average *μ* with noise level *ɛ*. Because the variance *σ*^2^(*E*) of the signal is also affected by changes in 〈*τ*〉 we scaled *ɛ* (see electronic supplementary material, note S6) to make sure the variance of the signal is comparable between simulations with different 〈*τ*〉 (figure 5*a*) and the analysis of the model results can focus on effects of the approximate period 〈*τ*〉 and variance of the synthetic signals *σ*^2^(*E*) similar to the previous sections. We synthesized 1000 random environmental signals for which we numerically tested how many of the windows are large enough to allow establishment by organisms with growth rate *λ*. As the number of potential windows (i.e. time intervals where *E* < *T*_0_) is variable, i.e. many but short windows at short periods 〈*τ*〉 and few but large windows at larger periods 〈*τ*〉—we report on the fraction attainable windows (*f*_WoO_) as an indication for the probability of establishment in such environmental conditions.

The numerical results clearly show that period 〈*τ*〉 and variance *σ*^2^(*E*) of the environmental fluctuations have a similar effect on the ability of organisms to establish in a harsh environment as previous explorations with the stylized DMN signal (figure 5*b,d*). At too slow growth rates *λ* no establishment can occur as the windows are too short. Increasing the period 〈*τ*〉 (from blue to black to red lines in figure 5*b*) results in a higher fraction of windows that are attainable. At larger periods, more windows are attainable, and establishment can thus occur at lower growth rates. Increasing the variance *σ*^2^(*E*) on the other hand reduces the fraction attainable windows (from red to black to blue line in figure 5*d*; black lines in b and d are the same). Yet, some minimal level of variance is required because if the variance is too small it cannot create any potential windows at which *E* < *T*_0_. In this specific example, the low variance signal (*σ*^2^(*E*) = 0.25) results in episodic emergence as revealed by the especially high variability in the numerical results (red line in figure 5*d*). Establishment becomes highly unlikely at *σ*^2^(*E*) < 0.25 because no windows are occurring. Hence, a reduction in variance can result in a sudden drop in establishment probability (see also grey area in electronic supplementary material, figure S1). The results underline that studying how organisms can establish using DMN-signals gives good intuitive understanding of how the colour (i.e. temporal correlation) and magnitude of the environmental fluctuations determine the establishment probabilities in other more complex environments such as those constructed using OUN. Hence, we expect that these insights will also apply to more complex and composite natural environmental signals.

## 5. Discussion

Studies about WoOs have so far mainly focused on developing mechanistic understanding about sudden establishment events observed in various ecosystems under harsh conditions (e.g. [4,7,8]). These studies pointed out that sudden emergence of stable occupancy can be explained as critical transitions, where establishment thresholds are transgressed due to variability of the environmental conditions [5,7]. Using hindcasts, the origins of the establishment events could indeed be linked to infrequent short time intervals of benign conditions—the so-called WoOs—underlining the validity of these mechanistic insights [7,8]. In this study, we developed a theoretical and statistical framework furthering our conceptual understanding of this type of noise-induced transitions. Moreover, this framework allows us to forecast the probability of future establishment events based on both the period and variance of the environmental variability in relation to the intrinsic timescales over which the organisms develop tolerance to the environment. Finally, we can apply these insights to better understand ecosystems responses to climate change, or shape management and restoration practices to kickstart these ecosystems. As we have shown that the stylized DMN signals capture the qualitative behaviour of other more complex signals well, we further discuss these three aspects based on insights from DMN signals.

### 5.1. Windows of opportunity as a specific class of noise-induced critical transitions

**Figure 6.**
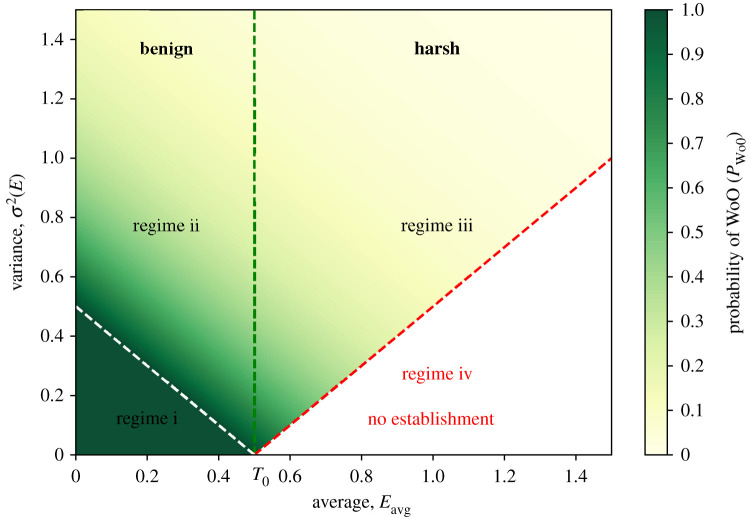
The interactive effects of environmental average and variance on the occurrence of WoO. The four regimes within the parameter space are indicated: in regime i noise is neglectable; in regimes ii and iii noise (i.e. temporal variability) is the relevant factor affecting establishment success; in the benign regime ii variability destroys opportunities; in regime iii noise can open up opportunities despite harsh average conditions; regime iv is unattainable. The green–yellow colour scheme depicts the occurrence of WoOs at the landscape-scale which is the ecological response to the interactive effects of environmental average *E*_avg_ and variance *σ*^2^(*E*). Note that no WoOs are present in regime iv. By default 〈*τ*〉 = 10 and *λ* = 0.05.

Formally, the concept of WoOs dealt with noise-induced critical transitions (i.e. ‘N-tipping', in [25,26]). Although not explicitly mentioned as such in earlier work (e.g. [4,7]), this notion explains the typical dynamics observed where thresholds need to be transgressed to allow establishment. However, noise-induced transitions related to WoOs exhibit specific characteristics that set them apart from the common notion of noise-induced transitions:

(1) In this work, we considered noise solely in the independent variable (i.e. parameter noise). Noise is usually—but not exclusively—considered to affect the dependent variable (i.e. demographic noise) which means variability is in the biological state (see e.g. Ashwin *et al*. [25] and Bonciolini *et al*. [26] and references therein).
(2) The WoO concept only focuses on the transition towards establishment of the organism (or emergence of an ecosystem); however, most theoretical studies about noise-induced transitions deal with both emergence and extinction transitions at the same time (e.g. Ashwin *et al*. [25], Bonciolini *et al*. [26] and Dakos *et al*., [27] and references therein) e.g. leading to back and forth ‘flickering’ between the two states (e.g. [27]). Therefore our work links better with the recently identified concept of phase tipping or partial tipping (P-tipping) in which a transition can only occur at a certain phase in the trajectory [28,29]. As ecosystems are usually more complex and multidimensional, the stressors limiting establishment (i.e. emergence) can be very different from the stressors responsible for their collapse (i.e. extinction). Therefore, it is reasonable to split the attention to develop appropriate mechanistic insights related to the different transition trajectories (i.e. emergence versus extinction transitions). There remains, however, scope to further develop the current framework to understand extinction transitions (see electronic supplementary material, note S3).
(3) The noise needs to be coloured because without any non-negligible temporal autocorrelation no WoO can exist. Then, the system remains locked in the unoccupied state. More specifically, for organisms to be at criticality (i.e. minimal requirements for establishment) the timescales of both the tolerance development by the organism and the environmental variability need to be proportional (following from equation (2.1)). That also means, that if noise whitens (i.e. the period shortens), the opportunities for establishment eventually collapse to a much smaller range of parameter space (regime i in figure 6). The fact that especially red noise and other temporally correlated signals (i.e. including periodic signals, such as the tidal, spring neap and seasonal cycles) are import drivers of critical transitions has only recently been recognized [22,30]. Hence, this special class of noise-induced transitions is likely to be common as such temporally correlated signals forcing ecosystem dynamics are widespread.

Despite the essential role of noise for creating WoOs in harsh environments, it has a fundamentally dualistic effect on establishment dynamics. In fact, in benign environments variance *σ*^2^(*E*) is destroying opportunities for establishment as it inhibits establishment in part of the time, even if the organism's initial tolerance *T*_0_ is above average conditions (regime ii in figure 6). Yet, at the same time temporal variability can also greatly expand opportunities into otherwise unattainable environments (regime iii in figure 6), as is the focus of this study. In these harsh environments (*T*_0_ < *E*_avg_), it is essential that the variance *σ*^2^(*E*) is large enough to be able to create benign windows. When the variance *σ*^2^(*E*) is too small, no windows are created at all (regime iv in figure 6). Only under the right conditions coloured noise—and other temporally correlated environmental signals—can open opportunities (regime iii in figure 6) that otherwise remain locked in a vacant stable state. Hence, temporally correlated signals can be responsible for widening the parameter space where organisms can transgress a tolerance threshold to establish and thrive (regimes ii and iii in figure 6).

### 5.2. From individual establishment thresholds to landscape-scale patterns

The consequence of the ‘unlocking' of alternative states due to environmental variability is that the ecosystem's response to changing environmental conditions is no longer discontinuous, as is at the individual level, but becomes continuous at the population or landscape scale. This means that, although the occurrence of a benign window might be a low probability event, establishment inevitably happens once the window of opportunity opens (in regions ii and iii in figure 6). If at that time plenty of propagules are present a sudden large-scale colonization event occurs transitioning large parts of the suitable landscape from one to the other state. Such rare episodic tree recruitment events have for instance been reported for tropical trees with variable rainfall conditions [4]. In case the presence of propagules is limited the successful establishment occurring during the WoOs can be the initiation of patch dynamics of bimodal patterns. These have been observed in a range of terrestrial and marine ecosystems [31,32]. Hence, the agreement between observations and our theoretical framework suggests that this class of noise-induced critical transitions is likely to be applicable to these ecosystems and might be for a larger range of ecosystems for which similar individual-level thresholds are reported or suggested.

### 5.3. Implications for management, restoration and climate change

**Figure 7.**
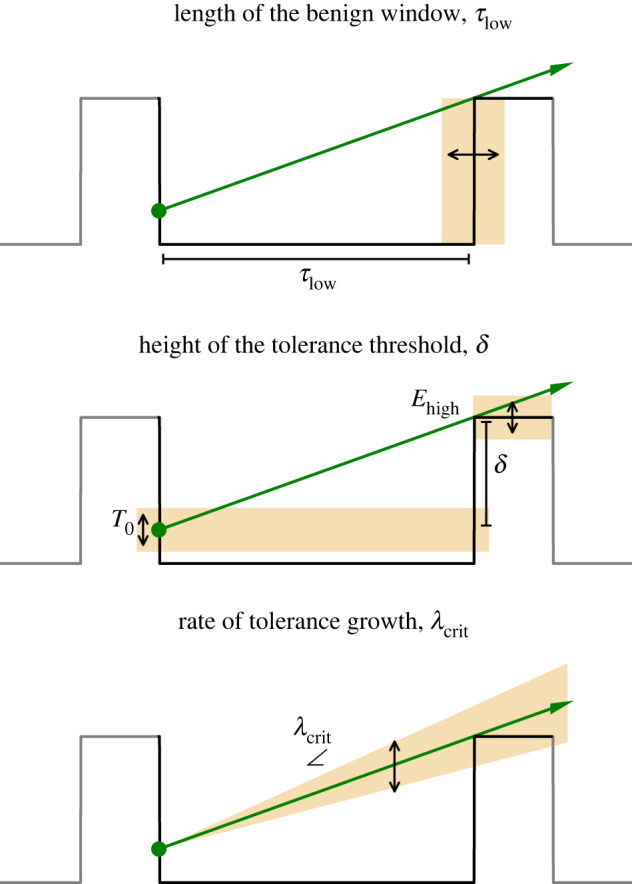
Summarizing overview of the three main options that affect establishment success.

The inherent stochastic nature of establishment dynamics can give the false impression that management or restoration cannot be fruitful. It has been argued that large-scale restoration efforts are needlessly expensive because organisms can recover by themselves without interventions. It only requires sufficient time and patience, as for example has been put forward for the establishment of bivalve beds [33] and seagrasses [34] in a soft-sediment environment where these ecosystems recovered naturally decades after landscape-scale loss. Given sufficient time, the entire feasible parameter space will eventually see establishment happen. However, our results also suggest that management actions can be taken to overcome establishment thresholds and speed up ecosystem recovery. In cases where stage- or density-dependent tolerance determines establishment success, the required minimal hospitable window (WoO) is essentially determined by the ratio of required tolerance gain to survive and the rate at which the organism develops this tolerance (equation (2.1)). This relationship leads to the insight that there are three basic options to affect establishment success (figure 7): (i) changing the length of the benign window*τ*_low_; (ii) changing the height of the tolerance threshold (*δ*) and (iii) changing the rate of tolerance growth (*λ*).

By prolonging the time window of benign conditions *τ*_low_ the establishment chances will be improved. However, changing the return time of the adverse condition might be the most difficult of the three options to achieve as the period is often set by regular environmental cycles, such as the season or tides. Attenuating the environmental condition is a more straightforward option to lower the tolerance threshold *δ* that needs to be overcome by the organisms. Especially attenuating high adverse conditions *E*_high_ can be a management option to create favorable conditions for establishment. Similarly, many species engineer their environment [35] e.g. by creating stress attenuating structures such as canopies [31,36]. It has recently been shown that structures mimicking these canopies can boost restoration success in coastal ecosystems [37]. Alternatively, the initial tolerance *T*_0_ can be boosted e.g. by skipping the most vulnerable life-stage by transplanting or introducing older more tolerant individuals which is a commonly used approach [36]. Improving growth rate also offers possibilities for management interventions by e.g. reducing local stressors or providing ample supply of otherwise limiting nutrients and other resources [38,39].

A somewhat counterintuitive, but important, conclusion of our study is that mere damping of environmental variance might be counterproductive, as it can reduce the occurrence of the benign conditions. For instance, natural water level fluctuations have been shown to be essential for creating WoOs [13]. Restoring the natural variability might therefore be the best strategy in some systems to obtain natural patterns of recruitment. Similarly, climate change can have sudden unexpected effects if increased variability suddenly opens otherwise unattainable environments (e.g. from regime iv to iii in figure 6). Thus, whatever the strategy chosen, a basic understanding of the intrinsic timescales of the environmental fluctuations is at least as important as developments in average conditions if one wants to be able to better predict ecological responses to ecological management or climate change.

Predicting the possible outcome of climate change and restoration efforts critically depends on appropriate prognostics of both system characteristics (e.g. period and variance of environmental variability, unidirectional changes in drivers) and characteristics of the organisms, most notably their ontogenetic range of tolerance and their vital parameters. Intervening in the basic parameters of the system of interest may alter the outcome of restoration profoundly. Understanding for example how temperature affects growth rates, might be key to explaining why ecosystem responses to a changing climate can be unexpectedly nonlinear. The theoretical framework we developed here moves away from the strict dichotomous view on critical transitions and can therefore better inform societal debate on the appropriateness of interventions.

## Data Availability

The code and generated synthetic data are made accessible via the NIOZ DAS open repository (https://doi.org/10.25850/nioz/7b.b.3c).
